# Deep Learning Based Early Detection Framework for Preliminary Diagnosis of COVID-19 via Onboard Smartphone Sensors

**DOI:** 10.3390/s21206853

**Published:** 2021-10-15

**Authors:** Hayat Khaloufi, Karim Abouelmehdi, Abderrahim Beni-Hssane, Furqan Rustam, Anca Delia Jurcut, Ernesto Lee, Imran Ashraf

**Affiliations:** 1 LAROSERI Laboratory, Department of Computer Science, Faculty of Sciences, Chouaib Doukali University, El Jadida 24000, Morocco; Hayat.khaloufi@gmail.com (H.K.); karim.abouelmehdi1@gmail.com (K.A.); abenihssane@yahoo.fr (A.B.-H.); 2 Department of Computer Science, Khwaja Fareed University of Engineering and Information Technology, Rahim Yar Khan 64200, Pakistan; furqan.rustam1@gmail.com; 3 School of Computer Science, University College Dublin, Belfield, Dublin 4, Ireland; 4 Department of Computer Science, Broward College, Broward County, FL 33332, USA; elee@broward.edu; 5 Department of Information and Communication Engineering, Yeungnam University, Gyeongsan 38541, Korea

**Keywords:** COVID-19 prediction, smartphone sensors, prediction framework, deep learning

## Abstract

The COVID-19 pandemic has affected almost every country causing devastating economic and social disruption and stretching healthcare systems to the limit. Furthermore, while being the current gold standard, existing test methods including NAAT (Nucleic Acid Amplification Tests), clinical analysis of chest CT (Computer Tomography) scan images, and blood test results, require in-person visits to a hospital which is not an adequate way to control such a highly contagious pandemic. Therefore, top priority must be given, among other things, to enlisting recent and adequate technologies to reduce the adverse impact of this pandemic. Modern smartphones possess a rich variety of embedded MEMS (Micro-Electro-Mechanical-Systems) sensors capable of recording movements, temperature, audio, and video of their carriers. This study leverages the smartphone sensors for the preliminary diagnosis of COVID-19. Deep learning, an important breakthrough in the domain of artificial intelligence in the past decade, has huge potential for extracting apt and appropriate features in healthcare. Motivated from these facts, this paper presents a new framework that leverages advanced machine learning and data analytics techniques for the early detection of coronavirus disease using smartphone embedded sensors. The proposal provides a simple to use and quickly deployable screening tool that can be easily configured with a smartphone. Experimental results indicate that the model can detect positive cases with an overall accuracy of 79% using only the data from the smartphone sensors. This means that the patient can either be isolated or treated immediately to prevent further spread, thereby saving more lives. The proposed approach does not involve any medical tests and is a cost-effective solution that provides robust results.

## 1. Introduction

During the last two decades, several pandemics broke out and challenged the capacity and claim of health systems and medical advancements. SARS (Severe Acute Respiratory Syndrome), Ebola, and MERS (Middle East Respiratory Syndrome) led to the deaths of hundreds of thousands of humans, besides other financial and economic losses. Unlike these pandemics, COVID-19 has emerged as a dangerous, quickly spreading, and lethal pandemic that has affected the whole world, and caused devastating economic and social disruption [1].

As COVID-19 continues to spread throughout the world, countries are facing incredibly difficult challenges in combating this disease, in particular through reducing its spread and ensuring access to adequate medical treatment to all the patients with severe symptoms [2,3]. Fortunately, at the beginning of 2021, many vaccines have been granted emergency use authorization and their rollout started in countries across the world, despite the fact that their ability to prevent transmission and the duration of immunity is verified only through ongoing wide-scale use. Moreover, a recent research report from CDC (Center for Disease Control and Prevention) [4] indicates that medical experts raise doubts about vaccines’ immunity being effective after six months. Consequently, strict behavioral measures, such as the wearing of face masks and maintaining physical distancing, remain recommended for controlling and curbing the spread of COVID-19. In addition, the mutation of the virus responsible for COVID-19 is a real threat to the efficacy of vaccines.

**Figure 1:**
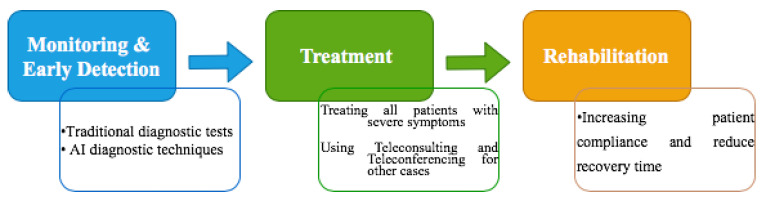
Three phases for healthcare systems in the battle against COVID-19.

To address key challenges related to COVID-19, the tech industry is increasingly seeking for repurposing technologies that have been used recently such as ubiquitous connectivity and big data to suit the needs of COVID-19–related healthcare services. Advances in the IoT (Internet of things), AI (Artificial intelligence) and cloud computing together offer an unprecedented opportunity to improve healthcare systems [5], thereby minimizing harmful effects on human health and saving countless lives. However, given the severity of COVID-19, putting that bold promise to the test, China, where the virus was first detected in late 2019, has effectively integrated technology in the governments’ health response systems to not only combat the ongoing outbreak of coronavirus but also guarantee the continuation of normal daily life. Managing the pandemic for reducing its impact and continuation of normal daily life requires an effective response. In addition, for reinforcing the prevention and control measures, specific actions must be undertaken in three main phases: monitoring and early detection, treatment, and rehabilitation phase, as illustrated in Figure 1.

**Phase 1: Monitoring and Early Detection**

The overall aim of this phase is to collect and interpret as much information as possible regarding COVID-19 signs and symptoms (such as fever, dry cough, headache, etc.) for early alerting and reporting while identifying and controlling the source of infection to prevent further spread. Several diagnostic tests for detecting COVID-19 are available so far. These tests are based on many techniques, including NAT (Nucleic Acid Test) which is a significant technique based on detecting the virus that can cause COVID-19 disease in blood [6], RT-PCR (Reverse Transcription Polymerase Chain Reaction), accepted by scientists and medical staff as a robust and well-documented technique [6] and CT (Computed Tomography) scan which is regarded as a fast, effective, and functional routine imaging tool for detecting the severity and degree of the lung inflammation [7].

Authors in [8] found that the sensitivity of CT for COVID-19 infection was 98% compared to RT-PCR testing sensitivity of 71%. However, several current studies [9,10] announce that chest CT scans are not recommended for routine screening as radiological findings in COVID-19 are not specific [11]. In Morocco, the Ministry of Health said that techniques used to detect the DNA of the Coronavirus (COVID-19) are the most reliable. Nevertheless, spending hours waiting in the clinic or hospitals for the examination, whatever the technique used for the diagnosis of the COVID-19 virus, thousands of patients are at high risk of cross-infection with other patients. It can lead to the widespread of COVID-19 and cause an unwanted burden for the medical system that is already on the edge of its limits.

**Phase 2: Treatment**

The treatment phase aims at ensuring that all cases detected are treated in a timely and effective manner while putting in place an efficient package of measures for isolating close contacts and putting them under medical observation, supporting front-line staff and individuals involved in this vital response. It is therefore crucial that the decision to initiate treatment should only be made after assessing the severity of COVID-19 symptoms. Patients with severe symptoms, including those who are required to use an oxygen mask must be admitted to the hospitals. Treating all patients with severe symptoms is a serious concern owing to the limited health system resources, medical supplies, and drugs. However, if detected early enough, the disease can be effectively treated and the burden on the hospitals and emergency rooms can be alleviated. In this case, the use of current technologies such as medical teleconsulting and teleconferencing is strongly recommended.

**Phase 3: Rehabilitation**

In the rehabilitation phase, appropriate safeguards must be followed as well as putting in place mitigation strategies and ensuring effective coordination in the short term to increase patient compliance and reduce the recovery time. However, there are already signs that COVID-19 may require a longer period of time for some patients to get a full recovery. The timing and extent of recovery depend crucially on the severity of symptoms as well as the effectiveness of treatment and how long it is performed.

Spending a long time in the hospital can risk the loss of muscle mass, but more seriously, the muscle will take time to build up again. Some patients will need longer rehabilitation techniques, such as physiotherapy to walk again. The report from the WHO (World Health Organization (WHO) published in February says that patients with a severe or critical case of COVID-19 can expect a median recovery time of three to six weeks while patients with mild symptoms will usually recover within one to three weeks [12]. From that perspective, several factors, including the huge demand for highly responsive, more efficient, and intelligent detection methods, clearly indicate that responding to the first phase is undoubtedly the most significant for containing the COVID-19 outbreak. The primary reason is that the currently available solutions are time-consuming and prone to late detection with possible errors. On the other hand, medical devices such as medical detection kits are used to detect coronavirus outbreaks. However, the potential and limits for its accuracy for detecting COVID-19 are still being assessed. In addition, these devices are costly and required installation for diagnosis. In this regard, this study makes the following contributions:

- A new framework is developed offering a preliminary diagnosis tool for COVID-19 using the data collected from individual users complemented by a machine learning approach.
- The framework is implemented by developing a smartphone app where the data are collected as the self-reported questionnaire. In addition, several smartphone embedded sensors are used for data collection as well such as thermometer, microphone, etc.
- A customized artificial neural network (ANN) is proposed for predicting COVID-19 based on the collected data. Experiments are performed using different sets of features to highlight the importance of critical feature selection. Results are discussed with respect to precision, recall, F1 score, as well as computation time and cross-validation.

The remainder of the paper is divided into five sections: Section 2 describes several related studies including the latest AI-based approaches and applications applying machine learning models for predicting COVID-19. Characteristics and architecture design of the proposed system for preliminary diagnosis of COVID-19 are carefully explained in Section 3. It also describes in detail the database used for training and validation of the algorithm while providing an overview of analytical methods adapted to construct the proposed machine learning model. The results and discussion are given in Section 4. The last section summarizes the work, highlights the key limitations, and highly recommends the integration of fog and cloud into the proposal for open research directions to increase the learning potential of the proposed AI system.

## 2. Literature Review, Background and Motivation

Around the globe, governments, health authorities and personnel in the health field are embracing stronger international cooperation to help combat the COVID-19 pandemic, protect citizens and accelerate the return of everyday life. New technologies including big data, IoT and AI are being rushed out in an attempt to save lives through early detection of emerging global health threats. It is in this spirit of cooperation that Apple and Google collaborate on an opt-in Bluetooth-based proximity contact detection API for iOS and Android. This solution allows for identifying and alerting people who may have come into contact with an infected person [13].

Indeed, findings in [14] indicate that contact tracing apps can be effective in reducing infection rates, even when just 60% of the population adopts them. In the same context, Morocco’s Ministry of Health launched the COVID-19 tracking application “Wiqaytna”, “our protection” in Arabic, to help curb the spread of the coronavirus in the country, by notifying participants about possible exposure, through someone they have been in contact with during the period of the last 21 days and who has subsequently been positively diagnosed. Following the notifications, teams from the Ministry of Health will assess the exposure risk and, if necessary, intervene to quarantine the notified suspected case [15]. Although this solution has continued to attract considerable national and international attention, there is still a need for an affordable and easy-to-use diagnostic tool to diagnose the preliminary results of the coronavirus disease and confirm suspicions.

### 2.1. Existing AI-Based Medical Imaging Techniques for COVID-19 Diagnosis

More recently, there has been an increasing interest in diagnosing COVID-19 using AI-based medical image analyses. The literature surveyed revealed that these approaches are leveraging analysis of either X-ray [16,17,18] or CT Scan [19,20,21] images. Furthermore, AI-based medical imaging assessment is seen as one of the promising techniques that can diagnose COVID-19 in much less time with higher accuracy. Indeed, the sensitivity of CT-based diagnosis has been reported as high and significantly better than RT-PCR can offer (95% confidence interval: 91–96%); however, specificity is on the low side (95% confidence interval 26–50%) [22]. However, while these techniques might lift some of the heavyweights of the radiologists’ shoulders, they still require a visit to an adequately equipped clinical setting which may increase contamination risks for the staff.

Self-reported symptoms during the COVID-19 pandemic have been used to train AI-based models to identify possible COVID-19 infection. Although studies based on early self-reported symptoms, such as [23,24], are widely used for identifying COVID-19 cases, no validated tool exists for surveying COVID-19 infection in the general population. Indeed, COVID-19 data are gathered in many countries to understand the pandemic and be prepared for the future. For example, Ref. [25] performs COVID-19 diagnosis using short-duration acoustic smartphone speech analysis. Moreover, authors in [26] provide a design study of an AI-enabled framework to diagnose COVID-19 using a smartphone. However, these models have only considered the peak of symptoms that is not suitable for the early detection of infection.

### 2.2. Using AI-Based Approaches for Preliminary Diagnosis of COVID-19

As the literature on the COVID-19 detection is not yet large, it is difficult to find correlations among the results reported due to various data used, adopted approaches and findings with respect to geographical location, ethnicity, living conditions, etc., innovative portable detection technologies that aim to identify certain major symptoms of this disease are discussed in this section. In fact, according to [27], as many as 98% of COVID-19 patients had a fever, 76% to 82% had a dry cough, and between 11% and 44% reported fatigue or myalgias. Other symptoms have been reported, such as headache, shortness of breath, sore throat, abdominal pain, and diarrhea. Based on the data at hand, the most common symptoms of the COVID-19 disease are fever, dry cough, fatigue, and headache.

The key symptom of the coronavirus is fever. Currently, temperature scan is the primary screening method for COVID-19 because of its easy detection and effective measurement. The smart thermometer provides an accurate value which serves as an important warning sign for possible patients. However, it does not track coronavirus cases or even enable preliminary diagnosis of COVID-19. In [28], a temperature fingerprint sensor has been proposed and experimentally implemented to predict the fever level using a smartphone’s touch-screen.

Cough is another main symptom found in the majority of COVID-19 patients; however, it is found in non-COVID-19 related diseases for age groups higher than 30. Consequently, it is an extremely challenging problem to discriminate the cough related to COVID-19 infection. The authors in [28,29] provided a comprehensive overview of different types of cough using a smartphone-microphone chipset. Similarly, Ref. [30] developed an AI-based preliminary diagnosis tool for COVID-19 using the cough sound via a mobile app. However, given the difficulty of getting cough data due to close contact requirements with the patient, the data were gathered only from a small number of patients. Despite the promising results of the study, a large-scale collection of labeled cough data is needed to gauge the generalization capability of this app and provide more accurate results. In [31], authors proposed the use of captured images and videos from a camera to detect human fatigue in different environments via human–gait analysis. Furthermore, in two other studies [32,33], the authors used onboard inertial sensors such as accelerometer and gyroscope, in order to detect fatigue levels.

The ’Migraine Buddy’, a free app created by Singaporean health data company Healint, claims to predict migraines with 90 percent accuracy [34]. There is an increasing trend for apps to use big data for helping consumers manage their health issues by asking them to guess the trigger for a particular headache. Furthermore, iHeadache [35] is also a free comprehensive electronic headache diary to determine what kind of headaches the user has. It was originally developed for the iPhone and BlackBerry and now it is also available online. Moreover, in [33], authors utilized the camera sensor and inertial sensors to predict the level of human- headache via neck posture monitoring. Despite the availability of such mobile apps and online services, gaps with the existing research require in-person visits to hospitals for proper investigation and tests. Such visits put both visiting persons and medical staff at a serious risk of infection. As a result, the infection in medics can lead to further scarcity of medical care services and increased medical distress. This could be identified as a fundamental limitation.

Another key limitation of the previously proposed approaches is that such approaches are not developed primarily for COVID-19 but can be used to identify certain symptoms which are similar in COVID-19 and other diseases such as cough, headache, etc. Furthermore, they do not address the issue of analytics completely. Furthermore, important symptoms related to COVID-19 patients are not considered fully such as fatigue, cough, fever, etc. Moreover, while there has been a movement towards AI-based solutions in the medical sector, a large gap exists when it comes to applying innovative machine learning models for automating the preliminary diagnosis of COVID-19. To bridge this gap and overcome the above-described problems, this study presents a novel framework that leverages smartphone built-in sensors for the preliminary diagnosis of COVID-19.

## 3. Proposed Framework for COVID-19 Diagnosis

To combat the increasing number of patients efficiently, there is a great need for further automation and advanced machine learning-based solutions that can take into account the main symptoms found in confirmed COVID-19 patients. It is in this context that this work provides an AI engine-powered mobile app-based solution that utilizes smartphone onboard sensors for tele-testing and preliminary medical diagnosis of COVID-19. The novelty of the proposed model comes from the perspective that it is more efficient, reliable, and risk-free to use several smartphone sensors considering multiple symptoms than considering only one or two symptoms as the state-of-the-art approaches do. Thereby, the proposed approach offers several benefits. First, the idea of using the smartphone embedded sensors is innovative where the thermometer and microphone are used to record the temperature and coughing patterns of individual users. The users also fill the self-reported questionnaire which further assists in the diagnosis process. Secondly, the users are not required to visit any hospital and medical experts which helps to constrain the pandemic as visits to hospitals or other diagnostic centers and transportation involve a higher risk of COVID-19 infection. Thirdly, the diagnostic results from the proposed framework help to have in-time treatment through precautions and medicine. Since the framework provides the preliminary diagnosis, it can be used for taking precautionary measures which reduce the probability of COVID-19 infection.

Additionally, the proposal has been specifically designed to offer a cost-effective, timely, and, most importantly, safe tool for monitoring and detection of COVID-19 and thus enabling a better assessment of the control of the rampant spread of the pandemic by allowing accurate preliminary diagnosis of infection in a large number of patients. Something that should not be neglected is that each of the COVID-19 symptoms could differentiate among other conditions including influenza symptoms, allergies symptoms, and cold symptoms. In this respect, it should be pointed out that the framework needs to discover the level of each symptom based on the measurements of embedded sensors.

### 3.1. Architecture Design and Overview of the Data Collection App

**Figure 2:**
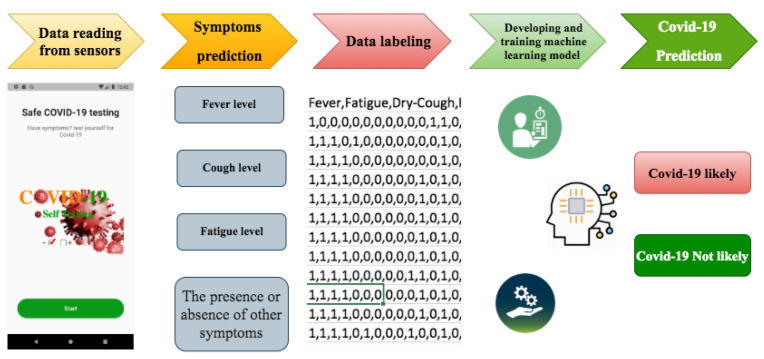
A diagrammatic representation of the proposed system for predicting COVID-19.

**Figure 3:**
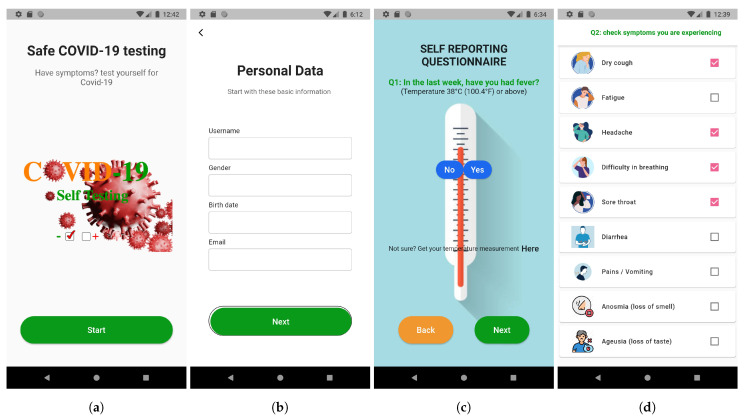
Snapshots of the app designed for data collection and prediction, (**a**) initial screen; (**b**) particulars screen; (**c**) temperature reporting; and (**d**) symptoms screen.

Data from the self-reported questionnaire and embedded sensors are sent to the fog node, where it is preprocessed and aggregated into three features: symptoms, age, and comorbidity. The model designed in this study is constructed as a set of layers. Figure 2 provides an overview of the proposed framework illustrating its components of data reading, symptoms prediction, and developing and training machine learning models. Data reading from sensors involves scanning temperature sensor measurements during fingerprint touching on the smartphone screen, recording sound measurements from the microphone during a series of cough, obtaining the accelerometer sensor measurements during 30-s sit-to-stand to analyze fatigue, and finally getting information about other symptoms that are pretty much restricted to yes or no answers. Symptoms prediction uses readings taken from smartphone sensors and provides the calculated symptoms level separately, and then store as a record input to the next layer. For symptoms collection, a smartphone app is designed with a series of screens, as shown in Figure 3.

Developing and training a machine learning model to predict COVID-19 comprises designing a custom feed-forward ANN (Artificial Neural Network). An ANN is seen as the most efficient and accurate deep learning approach that is ideally suited for health informatics and particularly for this model. ANN algorithms are a class of neural networks that are utilized for classification or recognition purposes as well as they are especially appropriate for processing time-series and other sequential data [36] such as text and signal measurements.

### 3.2. Data Description

The data used for this research are obtained from the Health Directorate of the Casablanca-Settat region. Only data from March 2020 to August 2020 are included in this analysis. During this period, 23% of 30,000 patients were reported as confirmed COVID-19 patients. The data contain information about patients’ symptoms, age, and comorbidity along with the severity of a patients’ illness.

**Figure 4:**
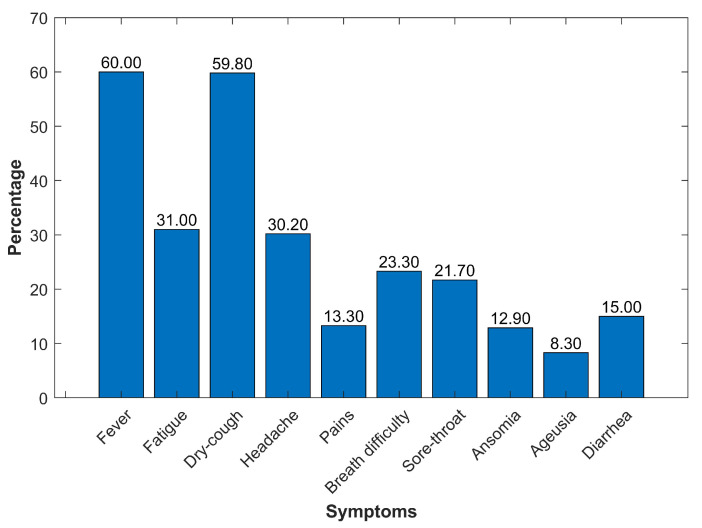
Distribution of symptoms found in COVID-19 patients.

As per the findings from the literature, these features are considered among the strongest predictors of confirmed COVID-19 cases. Findings from multivariate analyses indicate that age and comorbidities are simultaneously predominant risk factors associated with COVID-19 severity [37]. As described in Section 2 with reference to several works that COVID-119 patients experience different symptoms [27] such as fever, dry cough, headache, etc, to show the distribution of symptoms in the collected data using the developed smartphone app, Figure 4 shows the percentage of symptoms found in the patients. Based on data at hand, a meta-analysis of COVID-19 patients shows fever (60%) as the most common symptom, followed by dry cough (59.8%) and fatigue (31%). Other symptoms noted are headache (30.2%), difficulty in breathing or shortness of breath (23.3%), sore throat (21.7%), and diarrhea (15%). The least common symptoms are pains including vomiting (13.3%), anosmia (12.9%), and ageusia (8.3%). Clearly, fever, dry cough, fatigue, and headache are the most common symptoms in the data collected from Morocco for COVID-19 patients. Table 1 shows the explanation of the symptoms with respect to considered values.

Selecting a set of appropriate features that can make prediction accurate and eliminating those features that are irrelevant and can decrease the model accuracy have a significant impact on the efficacy of the resulting model. Having said that, correlation analysis has been exploited to identify relevant attributes from the dataset with a significant impact on the classification of COVID-19 diagnosis. Features’ correlation analysis is an extensively used technique to determine the relevance and similarity of attributes concerning the target class. High correlation (positive or negative) of a specific feature with data labels indicates the importance of a feature. On the other hand, a highly correlated variable should be considered and examined carefully. Simply put, it should not be included in data processing as it increases input size while not contributing to the prediction.

**Figure 5:**
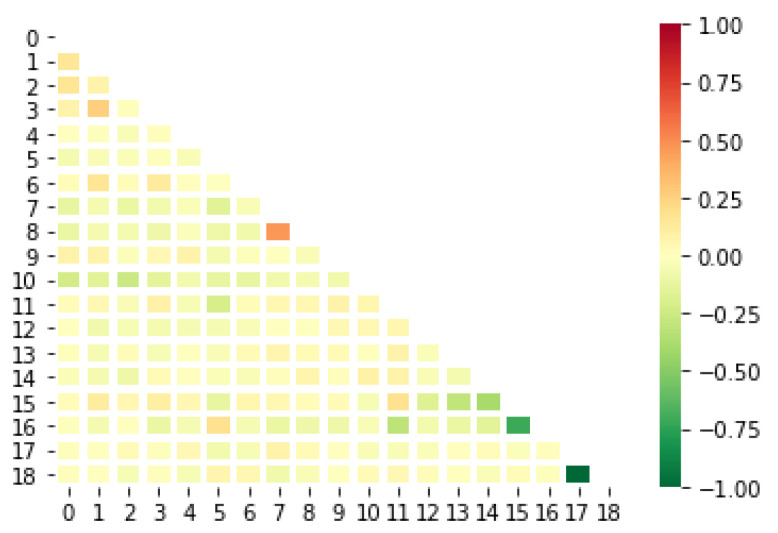
Scatter plot showing correlations between 19 selected features.

In this research, the correlation among independent features as well as between dependent and independent features has been carried out to determine the strong and consistent features. The features with high correlation (correlation higher than 0.65) have been discarded. The final set of features used as input of the machine learning algorithm in this study includes only 19 out of 24 features. These are fever (assigned the index 0), fatigue (index 1), dry-cough (index 2), headache (index 3), pains (index 4), difficulty-in-breathing (index 5), sore-throat (index 6), anosmia (index 7), ageusia (index 8), diarrhea (index 9), none symptom (index 10), none experiencing (index 11), age_0–9 (index 12), age_10–19 (index 13), age_20–24 (index 14), age_25–59 (index 15), age_60+ (index 16), gender female (index 17), and gender male (index 18). The indices are obtained by performing numerous training and validation procedures similar to calculating the optimization surface. A correlation plot for the final features used in this study is presented in Figure 5. Table 2 shows all the features gathered during the data collection process along with the number of negative and positive cases for each feature. One feature is country; however, owing to the fact that all cases studied here are from Morocco, the ’country’ feature is not added in Table 2.

To train and test the model, the cleaned data are split randomly into train and test subsets. However, as is well known that ensuring the practical viability of AI-based solutions is generally depending on the quantity and quality of the data. Therefore, the available dataset here is used to evaluate the practical feasibility of the proposed solution, while it is still insufficient to take full advantage of the AI system.

The used dataset consists of 1417 sample subjects including both COVID-19 positive and COVID-19 negative subjects with 1054 subjects for positive COVID-19 tests, and 363 subjects for negative COVID-19 tests, as shown in Table 3. The data are randomly divided into training 70% and testing 30% subsets.

### 3.3. Machine Learning Models and Data Analytics

The problem was formulated as a binary classification problem with COVID-19 likely as being a positive class and COVID-19 not likely as the negative class, based on a set of explanatory input features. Generally, there are two major types of classification problems: binary problems and multi-class problems—while in a binary problem, the given data-set is categorized into two classes, in a multi-class problem, the given data-set is categorized into several classes based on classification rules [38]. Thus, finding a model that can best predict the diagnosis of a COVID-19 infection (notated as 1 or 0, for the “positive” and “negative” classes) is the global aim of the binary classification. The collected data are normalized before feeding to training models. Machine learning models for the study are implemented in the Spyder environment (version, 4.0.1).

**Figure 6:**
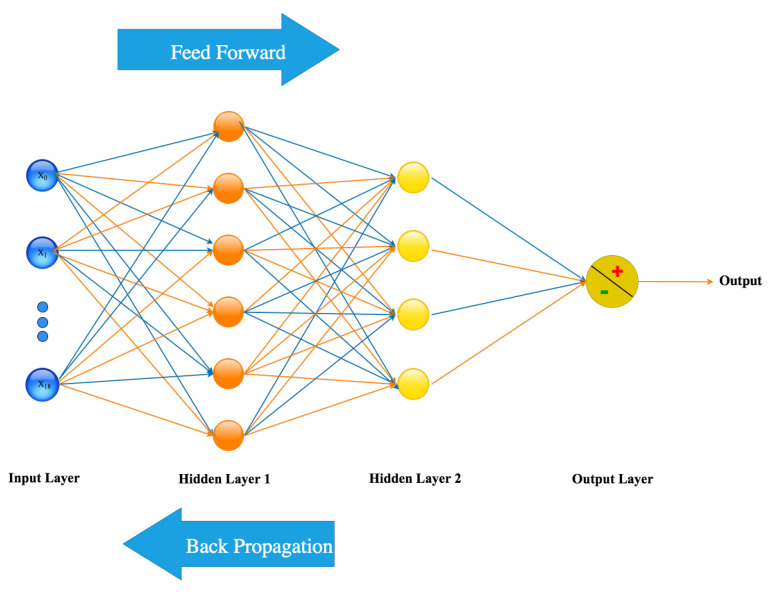
Diagram presenting the final architecture of ANN.

Several classification algorithms such as SVM (Support Vector Machine), RF (Random Forest), feed-forward ANN, LR (Logistic Regression), DT (Decision Tree), and K-NN (K-Nearest Neighbors) have been trained and tested to analyze their performance to find the highest numerical results. The ANN classifier has been selected as it is balanced in terms of accuracy, sensitivity, and F1-score. The architecture of the used ANN in this study is given in Figure 6. This architecture includes 19 inputs $(X_{0}$ to $X_{18})$, 6 hidden neurons in the first hidden layer, 4 hidden neurons in the second hidden layer, and 1 output neuron.

Achieving the optimized results is accomplished by fine-tuning several parameters of the ANN algorithm based on the cross-validation accuracy. Among these are the network model, network size, the activation function, learning parameters, and the number of training samples. Similarly, many important hyperparameters are optimized using the grid search function for achieving better accuracy with the machine learning models. A complete list of parameters and their values used in this study are given in Table 4.

### 3.4. Proposed Machine Learning Based Framework

**Figure 7:**
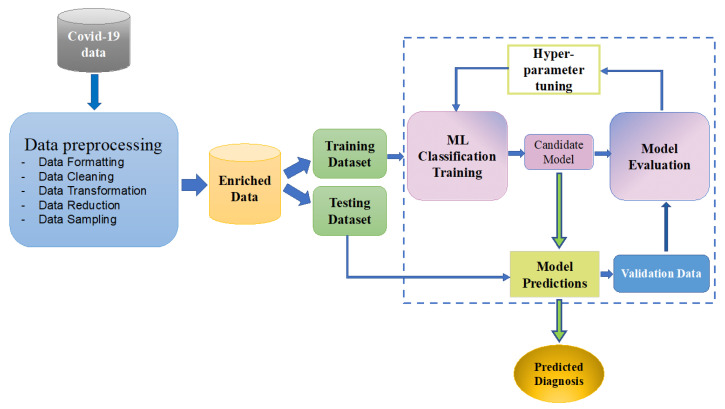
Work flow diagram of machine learning based framework for detecting the likelihood of COVID-19.

Figure 7 shows the workflow of the proposed framework for detecting the likelihood of COVID-19. Different data inputs are first preprocessed (formatted, cleaned, and sampled) before applying machine learning algorithms for training. Once the data are prepared, specific machine learning algorithms are applied for data modeling and validation to produce an optimized model. In this iterative process, we tested various algorithms until the classifier that performs the best is found.

In the proposed model, a feed-forward is used with 19 inputs and 1 output. The Sigmoid function is used as an activation function only in the last layer of the neuron model and the ReLU (Rectifier Linear Unit) function is used for the first three layers. The K-fold cross-validation method is used and optimal results are obtained at k = 10. The RMSProp is used as the optimizer due to its relatively better efficiency and flexibility. The number of neurons in the hidden layers and the number of iterations are tuned by several combinations of parameters where the training time of each combination is up to 120 iterations to obtain better statistics. The highest accuracy is obtained with 100 iterations, 6 hidden neurons in the first hidden layer and 4 hidden neurons in the second one. Furthermore, the decay of model loss by binary cross-entropy loss function versus the number of epochs has been investigated to rule out the possibility of over-fitting.

### 3.5. Performance Evaluation Metrics

Several well-known performance evaluation metrics are used to analyze the performance of machine learning models used in this study. For this purpose, accuracy, precision, sensitivity, specificity, and F1 scores are used with the following expressions:

(1) $$\begin{array}{c} \mathrm{Accuracy}=\frac{\left(\mathrm{TP}+\mathrm{TN}\right)}{\mathrm{TP}+\mathrm{TN}+\mathrm{FP}+\mathrm{FN}} \end{array}$$

(2) $$\begin{array}{c} \mathrm{Precision}=\frac{\mathrm{TP}}{\mathrm{TP}+\mathrm{FP}} \end{array}$$

(3) $$\begin{array}{c} \mathrm{Sensitivity}=\frac{\mathrm{TP}}{\mathrm{TP}+\mathrm{FN}} \end{array}$$

(4) $$\begin{array}{c} \mathrm{Specificity}=\frac{\mathrm{TN}}{\mathrm{TN}+\mathrm{FP}} \end{array}$$

(5) $$\begin{array}{c} F1\;\mathrm{score}=2\times \frac{\mathrm{Precision}\times \mathrm{Sensitivity}}{\mathrm{Precision}+\mathrm{Sensitivity}} \end{array}$$

where TP refers to true positive (correctly identified), TN to true negative (correctly rejected), FP to false positive (incorrectly identified), and FN to false negative (incorrectly rejected).

Accuracy measures the overall number of correctly classified samples out of total samples, precision (or positive predictive value) shows how many of the positively classified samples are actually positive, sensitivity (or recall) measures correctly identified actual positives, specificity (or selectivity) measures correctly identified actual negatives and F1 score is the harmonic mean of the precision and sensitivity [39].

## 4. Results and Discussion

Several experiments are performed in this study to analyze the classification accuracy of different machine learning algorithms and the proposed ANN model. Similarly, the influence of the different number of features is also studied to show the importance of the feature selection approach. The data are first processed to find the most important features that make the classification better. The aim is to find the best feature subset based on data characteristics without depending on classification models [40]. For this reason, filter-based feature selection investigates different data relations such as the relation between features and class (e.g., relevancy) and the relation among features (e.g., redundancy, and dependency). Relevancy relation measures the amount of shared information between features and the class [41]. Some features may have the same relevancy relation and do not add new information to discriminate the classes. These features are considered redundant and should not be considered. Redundancy relation measures the amount of shared information among features [41]. Another important feature relation is dependency. Dependency relation measures the membership degree of feature subset to class.

**Figure 8:**
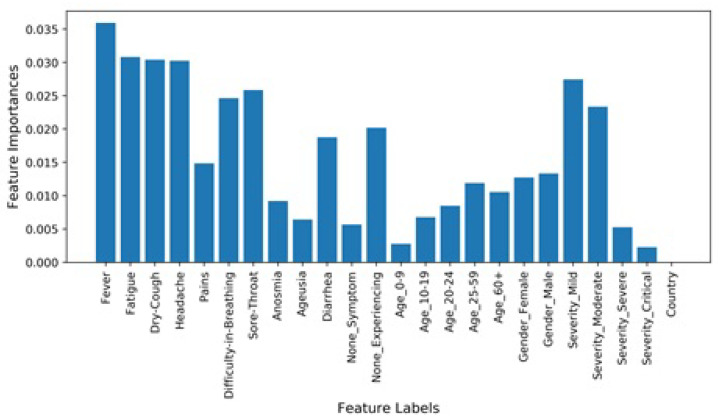
Feature importance based on a decision tree model.

**Figure 9:**
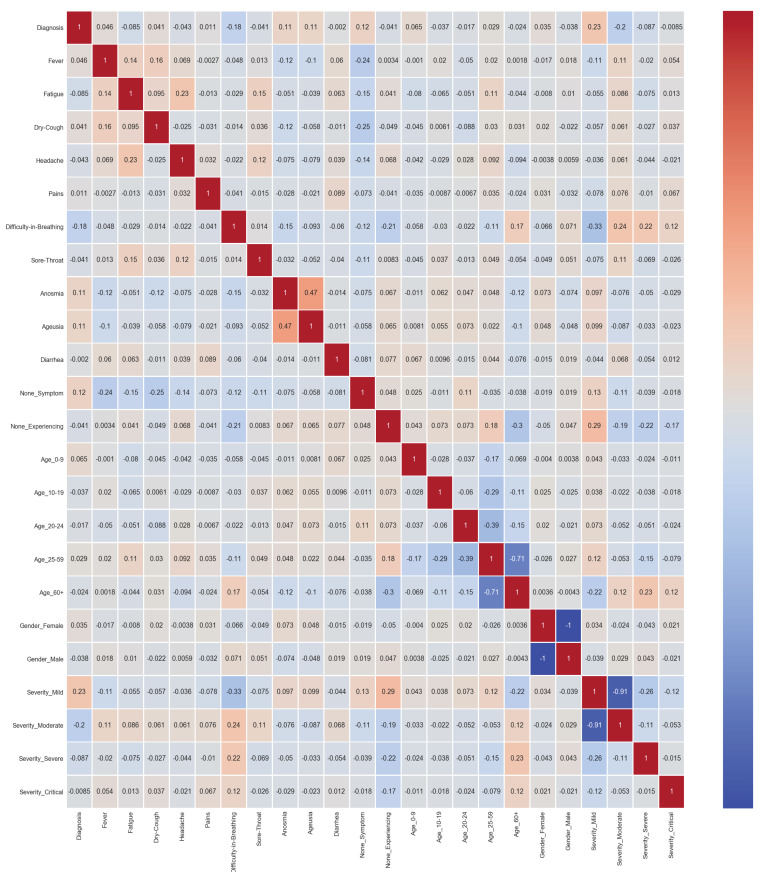
Correlation of all 24 features for the collected data.

A decision tree-based feature weighting has been carried out to determine the importance of the features for COVID-19 diagnosis. Feature importance with respect to its correlation to the target class is shown in Figure 8. It shows that ‘country’, ‘severity_critical’, and ‘age_o-9’ are the features with the least importance to diagnose COVID-19. Consequently, five of the twenty four features have been dropped. Based on feature importance analysis and literature findings [1,42], the 19 features used in this study are the main factors influencing the severity levels of COVID-19. A correlation plot for all 24 features has also been added, as shown in Figure 9.

To corroborate the importance of features, experiments are performed using all 24 features, 19 selected features, and 17 selected features. Features are selected based on the correlation analysis. Table 5 shows the results of models used in this study when trained and tested using a different set of features.

Results indicate that models perform better when used with 19 selected features as compared to using all 24 and 17 selected features, as shown as bold values in Table 5. From these results, it could be concluded that the selected 19 features provide the optimum results and further experiments are performed using the selected 19 features. Although a higher number of features are used in several classification problems to achieve high accuracy; however, increasing the number of features does not guarantee higher accuracy. For example, Ref. [43] shows that the performance of C4.5 and SVM is decreased as long as the number of features is increased. On the other hand, selecting critical/important features is important for enhancing the performance, as pointed out in [44]. The number of features depends on the domain, data, and nature of the problem as well. For example, for COVID-19 data, a large number of features are either not available or not appropriate. Consequently, a small number of important features are preferred for the problem at hand. Study [45] uses only 17 features for predicting the health of COVID-19 patients with a boosted RF model. Similarly, the authors make use of 14 clinical features to predict the COVID-19 severity in [46]. Keeping these findings in view, it can be stated that the selected 19 features can produce good classification accuracy considering the fact that a large feature vector is not available for COVID-19 data.

Results are further evaluated using several metrics including the accuracy, precision, sensitivity, specificity, and F1 score for each model with respect to each class. Table 6 shows the results for all models using selected 19 features. The given performance metrics are based on the mean confusion matrices from cross-validation. Results show that all models perform well when used with 19 selected features with the lowest accuracy of 0.71 from DT while the highest accuracy is achieved by the ANN, i.e., 0.79. The weighted average for the ANN is also the highest with 0.78, 0.79, and 0.78 for precision, recall, and F1 score, respectively. LR has a marginal difference in performance with a 0.78 accuracy core while weighted averages are 0.77, 0.78, and 0.71 for precision, recall, and F1 score, respectively. KNN, RF, and SVM achieve accuracy scores of 0.74, 0.75, and 0.76, respectively.

In addition to individual class accuracy, precision, and other performance evaluation metrics, averaged performance of each model is provided in Table 7, which suggests that ANN outperforms all other models in terms of accuracy, precision, and F1 score. Similarly, the sensitivity of the ANN is appropriate given the task at hand. The resulting sensitivity of the proposed algorithm is higher than specificity, which has been found acceptable from the point of view of the safety of the public and healthcare workers. In fact, sensitivity is particularly important in understanding the risk of false-negative testing [47]. Thus, the minimization of the false-negative factor is the property of interest due to the magnitude of risk from false-negative test results that will be substantial as testing becomes more widespread and the prevalence of COVID-19 infection rises.

The risk to individuals with false-negative results is mainly that they may relax physical distancing and other personal measures designed to reduce the transmission of the virus to others. These results also involve numerous other risks, including the delayed or lack of supportive treatment, lack of monitoring of infected individuals and their household, or other close contacts [48], which has direct implications for the success of efforts to curb the pandemic. Work will continue to incorporate improvements, as more training data become available.

Table 8 shows the number of TP, TN, FP, FN, as well as the number of WP (wrong predictions) and CP (correct predictions) for the experiments performed in this study. ANN shows superior performance with the highest 337 CP and lowest 89 WP, followed by LR with 331 and 95 number of CP and WP, respectively. The DT proved to be the worst performing model with the highest 123 wrong predictions for the task at hand. The performance of DT is poor on account of several factors. It classifies by step-wise assessment of a data, dealing one node at a time. It starts from root node towards the terminal node with two possibilities at each node. Consequently, several variables’ relationships are not learned by DT, which cause incorrect prediction in the test phase.

For further evaluation of the model’s performance, the computational cost is used as the evaluation parameter as well. Time is calculated for the model training and testing process. Table 9 shows the computational time of all the models used in this study. Results show that the ANN has higher computational time as compared to machine learning models, except for RF. It is because ANN training involves an iterative process and has 100 epochs which increase the computational time. RF takes 43.93 as it involves a large number of decision trees due to its ensemble structure. Other machine learning models have significantly lower processing time with KNN having the lowest time of 0.20 s.

The performance of the machine learning models is further confirmed using 10-fold cross-validation. The purpose is to show the significance of the proposed approach with respect to other models. Table 10 shows the cross-validation results with standard deviation for all the models used in this study. It shows that results of ANN are also better as compared to other models with 10-fold cross-validation as ANN achieves a mean accuracy of 79% with ±0.01 standard deviation.

In the light of the discussed results, it can be assumed that the proposed approach proves to be a significant tool that can prove the preliminary results for the diagnosis of COVID-19 probable cases. The novelty of the approach comes from the fact that it uses only a small amount of data obtained from the smartphone’s embedded sensors and helps to constrain the pandemic. Additional setup is not required for the proposed approach, which makes it a cost-effective solution as well. Additionally, relying only on the smartphone sensors data without the need to visit a medical expert physically is very helpful to contain the pandemic and helps to reduce the possible COVID-19 cases.

## 5. Conclusions and Future Work

Motivated by the urgent worldwide need to win the ongoing war against the COVID-19 pandemic, this paper presents an AI-based framework that enables preliminary diagnosis for COVID-19 using smartphone embedded sensors and self-reported questionnaires. The preliminary results demonstrate the efficiency and practical feasibility of the proposed solution. Results show that, using the proposed framework, an accuracy of 79% is obtainable for the preliminary diagnosis of COVID-19 using the ANN model. The proposed framework has several benefits such as a lack of need to visit hospitals or clinics physically for diagnosis, reduced risk of physical contact with probable COVID-19 patients, no cost for tests, and diagnosis using smartphone embedded sensors at any time and anywhere which can substantially reduce the unnecessary visits to hospitals and help to reduce the widespread of COVID-19.

Nonetheless, there are still several ways to extend the present study for taking full advantage of the AI system by incorporating several additional parameters. The available dataset is relatively small and lacks patients with other types of coronavirus. Further research is therefore required to investigate whether the classifier can distinguish between other types of COVID-19. One of the main elements of this system is the mobile application for smartphones that should discover the level of each symptom based on the measurements of embedded sensors. For instance, extracting a cough needs to record sound measurements from the microphone during a series of coughs; then, the measured signal will be classified and analyzed by an appropriate algorithm. However, the data at hand consider only the existence or the absence of each symptom and not the level or magnitude. Hence, data collection is still in progress for more comprehensive analysis. Careful coordination of fog and cloud components in the setup is highly advocated since employing fog computing could reduce the amount of data transferred to the cloud. The reduction of data becomes an even more significant aspect as the amount of data collected and streamed would rapidly increase.

These points bring up an interesting opportunity for future work. Given more adequate data, the performance and reliability of the proposed model are expected to be further improved and more robust. As a matter of fact, the middle-term goal is to use fog nodes not only to reduce the communication overhead between mobile devices (smartphones) and cloud storage but also to preserve the data privacy of the patient. Through this framework, signal measurements from the smartphone sensors are aggregated at the fog node and analyzed in the cloud, to predict the result of the disease, and further identify the grade of severity as either mild, moderate, severe, or critical. 

## Figures and Tables

**Table 1:** COVID-19 symptoms values for positive and negative cases.

Symptoms	Positive Value	Negative Value
Fever	Temperature ≥ 38	Temperature < 38
Fatigue (myalgia)	Yes	NO
Dry cough	Yes	NO
Headache	Yes	NO
Pains/Vomiting	Yes	NO
Difficulty in breathing or Shortness of breath	Yes	NO
Sore Throat	Yes	NO
Nasal congestion or Anosmia (loss of smell)	Yes	NO
Ageusia (loss of taste)	Yes	NO
Diarrhea	Yes	NO

**Table 2:** Characteristics and descriptions of the features used in this study.

Feature	Class	Total (1517)		COPVID-19 Nengative (363)		COVID-19 Positive (1054)	
		n	%	n	%	n	%
**Symptoms**							
Fever	True	832	(58.7%)	199	(54.8%)	633	(60%)
	False	585	(41.3%)	164	(45.2%)	421	(40.0%)
Fatigue	True	466	(32.9%)	144	(39.7%)	322	(30.6%)
	False	951	(67.1%)	219	(60.3%)	732	(69.4%)
Dry-Cough	True	830	(58.6%)	200	(55.1%)	630	(59.8%)
	False	587	(41.4%)	163	(44.9%)	424	(40.2%)
Headache	True	444	(31.3%)	126	(34.7%)	318	(30.2%)
	False	973	(68.7%)	217	(65.3%)	756	(69.8%)
Pains	False	185	(13.1%)	45	(12.4%)	140	(13.3%)
	True	1232	(86.9%)	318	(87.6%)	914	(86.7%)
Difficulty in Breathing	True	397	(28.0%)	151	(41.6%)	246	(23.3%)
	False	1020	(72%)	412	(58.4%)	808	(76.7%)
Sore-Throat	True	322	(22.7%)	93	(25.6%)	229	(21.7%)
	False	1095	(77.3%)	270	(74.4%)	825	(79.3%)
Anosmia	True	154	(10.9%)	18	(5.0%)	136	(12.9%)
	False	1263	(89.1%)	345	(95.0%)	918	(87.1%)
Ageusia	True	96	(6.8%)	8	(2.2%)	88	(8.3%)
	False	1321	(93.2%)	355	(97.8%)	966	(91.7%)
Diarrhea	True	213	(15.0%)	55	(15.2%)	158	(15.0%)
	False	1204	(85.0%)	308	(84.8%)	896	(85.0%)
No Symptom	True	63	(4.0%)	1	(0.3%)	62	(5.0%)
	False	1354	(96.0%)	362	(99.7%)	992	(94.1%)
**Basic information** The age of a tested individual is based on the WHO age group standard.							
Age_0-9	True	24	(1.7%)	1	(0.3%)	23	(2.2%)
	False	1393	(98.3%)	362	(99.7%)	1031	(97.8%)
Age_10-19	True	60	(4.2%)	20	(5.5%)	40	(3.8%)
	False	1357	(95.8%)	343	(94.5%)	1014	(96.2%)
Age_20-24	True	106	(7.5%)	30	(8.3%)	76	(7.2%)
	False	1311	(92.5%)	333	(91.7%)	978	(92.8%)
Age_25-59	True	920	(64.9%)	227	(62.5%)	693	(65.7%)
	False	497	(35.1%)	136	(47.5)	361	(34.3%)
Age_60+	True	304	(21.5%)	84	(23.1%)	220	(20.9%)
	False	1113	(78.5%)	279	(76.9%)	834	(79.%)
Gender Female	True	671	(47.4%)	161	(44.4%)	510	(48.4%)
	False	746	(52.6%)	202	(55.6%)	544	(51.6%)
Gender Male	True	746	(52.6%)	202	(55.6%)	544	(51.6%)
	False	671	(47.4%)	161	(44.4%)	510	(48.4%)
**The severity of a patients’ illness**							
Severity Mild	True	967	(68.2%)	181	(49.9%)	786	(74.6%)
	False	450	(31.8%)	182	(99.7%)	268	(26.4%)
Severity Moderate	True	396	(27.9%)	158	(43.5%)	238	(22.6%)
	False	1021	(72.1%)	205	(56.%)	816	(77.4%)
Severity Severe	True	45	(3.2%)	21	(5.8%)	24	(2.3%)
	False	1372	(96.8%)	342	(94.2%)	1030	(97.7%)
Severity Critical	True	10	(0.7%)	3	(0.8%)	7	(0.7%)
	False	1407	(99.3%)	360	(99.2%)	1047	(99.3%)
**Commorbidity**							
None Experiencing	True	1,189	(83.9%)	314	(86.5%)	875	(83.0%)
	False	228	(16.1%)	49	(13.5%)	179	(17.0%)

**Table 3:** Dataset count for training and testing data used in the study.

Dataset	COVID-19 Positive	COVID-19 Negative	Total
Total	1054	363	1417
Training set	732	259	991
Test set	322	104	426

**Table 4:** List and values of hyperparameters used for machine learning models.

Model	Hyperparameter	Hyperparameter Range
LR	Solver = liblinear, Penalty: l2, C: 3.0	Solver = liblinear, sag, saga, Penalty: l1, l2, C: 1 to 5
RF	n_estimators = 100, max_depth = 12, min_samples_leaf = 0.02	n_estimators = 10 to 200, max_depth = 2 to 50, min_samples_leaf = 0.01 to 0.05
DT	max_depth = 12, min_samples_leaf = 0.02	max_depth = 2 to 50, min_samples_leaf = 0.01 to 0.05
SVM	Kernel = Polynomial, C = 3.0, degree = 1	Kernel = Polynomial, linear, C: 1 to 5, degree = 1 to 5
KNN	n_neighbors = 2	n_neighbors = 1 to 5
ANN	Hidden layers = 2, optimizer = rmsprop, loss = binary_crossentropy, batch_size = 5, epochs = 100	Hidden layers = 2 to 5, optimizer = rmsprop, adam, SGD, loss = binary_crossentropy, batch_size = 5, 10, 15, epochs = 50, 100, 150

**Table 5:** Accuracy of different models based on different set of features.

Number of Features	LR	RF	DT	SVM	KNN	ANN
24	0.76	0.75	0.69	0.74	0.74	0.78
**19**	**0.78**	**0.75**	**0.71**	**0.76**	**0.74**	**0.79**
17	0.77	0.745	0.73	0.76	0.725	0.77

**Table 6:** The results of different classification algorithms trained in this work.

Model	Accuracy	Class	Precision	Recall	F1-Score
LR	0.78	0	0.74	0.13	0.23
		1	0.78	0.98	0.87
		Macro avg	0.76	0.56	0.55
		Weighted avg	0.77	0.78	0.71
RF	0.75	0	0.52	0.33	0.40
		1	0.80	0.90	0.84
		Macro avg	0.66	0.61	0.62
		Weighted avg	0.73	0.75	0.73
DT	0.71	0	0.44	0.50	0.47
		1	0.82	0.79	0.80
		Macro avg	0.63	0.64	0.63
		Weighted avg	0.72	0.71	0.72
SVM	0.76	0	0.73	0.10	0.18
		1	0.76	0.99	0.86
		Macro avg	0.75	0.54	0.52
		Weighted avg	0.75	0.76	0.69
KNN	0.74	0	0.48	0.40	0.44
		1	0.81	0.85	0.83
		Macro avg	0.64	0.63	0.63
		Weighted avg	0.72	0.74	0.73
ANN	0.79	0	0.60	0.43	0.50
		1	0.83	0.91	0.87
		Macro avg	0.72	0.67	0.69
		Weighted avg	0.78	0.79	0.78

**Table 7:** Performance evaluation metrics for models used in this study.

Model	Accuracy	Precision	Sensitivity	Specificity	F1 Score
LR	0.78	0.78	0.98	0.14	0.868
RF	0.75	0.796	0.896	0.33	0.843
DT	0.71	0.819	0.785	0.495	0.801
SVM	0.76	0.762	0.987	0.10	0.86
KNN	0.74	0.806	0.852	0.404	0.828
ANN	0.79	0.86	0.91	0.43	0.884

**Table 8:** Confusion matrix of classification algorithms used in this study.

Model	TP	TN	FP	FN	WP	CP
LR	317	14	90	5	95	331
RF	284	36	73	33	106	320
DT	249	54	55	68	123	303
SVM	313	11	98	4	102	324
KNN	270	44	65	47	112	314
ANN	306	45	59	30	89	337

**Table 9:** Computational time for models.

Model	Computation Time (s)
ANN	33.36
DT	0.55
RF	43.93
SVM	22.90
KNN	0.20
LR	23.69

**Table 10:** Results of 10-fold cross-validation for all models.

Model	Accuracy % (±Std. Dev.)
LR	74 (±0.01)
RF	72 (±0.02)
DT	67 (±0.04)
SVM	74 (±0.02)
KNN	73 (±0.02)
ANN	79 (±0.01)

## Data Availability

Not applicable.
